# Evolving Healthcare Quality in Top Tertiary General Hospitals in China during the China Healthcare Reform (2010–2012) from the Perspective of Inpatient Mortality

**DOI:** 10.1371/journal.pone.0140568

**Published:** 2015-12-01

**Authors:** Xie-Min Ma, Xiao-Hong Chen, Ji-Shan Wang, Gary H. Lyman, Zhi Qu, Wen Ma, Jing-Chen Song, Chuan-Kun Zhou, Lue Ping Zhao

**Affiliations:** 1 School of Public Health, Peking University Health Science Center, Beijing, People’s Republic of China; 2 National Institute of Hospital Administration, China National Health and Family Planning Commission, Beijing, People’s Republic of China; 3 Division of Public Health Sciences, Fred Hutchinson Cancer Research Center, Seattle, Washington, United States of America; 4 School of Public Health, University of Washington, Seattle, Washington, United States of America; Thomas Jefferson University, UNITED STATES

## Abstract

Healthcare reforms (HR) initiated by many countries impacts on healthcare systems worldwide. Being one of fast developing countries, China launched HR in 2009. Better understanding of its impact is helpful for China and others in further pursuit of HR. Here we evaluate inpatient mortality, a proxy to healthcare quality, in 43 top tertiary hospitals in China during this critical period. This is a hospital-based observational study with 8 million discharge summary reports (DSR) from 43 Chinese hospitals from 2010–2012. Using DSRs, we extract the vita status as the outcome, in addition to age, gender, diagnostic codes, and surgical codes. Nearly all hospitals have expanded their hospitalization capacities during this period. As of year 2010, inpatient mortality (IM) across hospitals varies widely from 2‰ to 20‰. Comparing IM of year 2011 and 2012 with 2010, the overall IM has been substantially reduced (OR = 0.883 and 0.766, p-values<0.001), showing steady improvements in healthcare quality. Surgical IM correlates with the overall IM (correlation = 0.60, p-value <0.001), but is less uniform. Over these years, surgical IM has also been steadily reduced (OR = 0.890 and 0.793, p-values<0.001). Further analyses of treatments on five major diseases and six major surgeries revealed that treatments of myocardial infarction, cerebral hemorrhage and cerebral infarction have significant improvement. Observed temporal and spatial variations demonstrate that there is a substantial disparity in healthcare quality across tertiary hospitals, and that these hospitals are rapidly improving healthcare quality. Evidence-based assessment shed light on the reform impact. Lessons learnt here are relevant to further refining HR.

## Introduction

Recent economic progress in China has stimulated the continuously increasing demand on the healthcare delivery system and lead the central government to launch multiple healthcare reforms since 1980’s[1, 2]. The most recent health care reform, starting from 2009, aims to improve public access to healthcare facilities by establishing government-subsidized insurance programs, regulating essential medicine list, improving referral system, expanding public health services, and piloting hospital reforms[3]. Building upon successes and failures of earlier health care reform efforts, the Chinese government has committed substantial financial resources into the current reform initiatives, leading to an impressive insurance coverage for over 95% of the population, and instituting various ways of delivering healthcare services to the vast population in diverse cities, counties and provinces of China[2]. Following the successful implementation of national health insurance program, recent efforts are shifted towards piloting reform efforts on “healthcare delivery organizations”, which is considered the most difficult tasks in the current healthcare reform. Beyond focusing only on insurance coverage as the primary evaluation criterion, the healthcare reformers are now interested in “patient-centered outcomes”, such as personal health status, patient satisfaction, reduction of financial burden, as well as the focus of this manuscript, the quality of healthcare[3]. Measuring quality of care by healthcare delivery organizations is not a trivial matter, and has to account for many factors, such as the nature of organizations, specialties of care, and sources of referral populations[4]. One of commonly used measurements is inpatient mortality (IM), despite its many limitations in measuring healthcare quality[5–11].

In this manuscript, we use IM as a proxy of healthcare delivery quality, to assess healthcare quality during these critical three years, from 2010 to 2012. The primary question of interest is if healthcare organizations are improving their healthcare quality, following the current healthcare reform. To address this question, we utilize a “Big Data” set of all discharge summary reports (DSRs) from 43 tertiary general hospitals, from January 1, 2010 to December 31, 2012. Our evidence-based investigation covers overall healthcare quality, quality of all surgeries, and treatment qualities of selected diseases and surgeries. Our results shed lights on recent progress in healthcare quality improvement in China and also suggest areas for further reform.

## Methods

### The Data Source

All 43 tertiary hospitals under considerations are large tertiary general public hospitals managed by local health bureaus, all of which are regulated by National Health and Family Planning Commission (NHFPC, http://www.nhfpc.gov.cn/), after merging Ministry of Health and Family Planning Commission. As part of the hospital management, all public hospitals are required to prepare a DSR on every hospitalization in their local health information systems, and batches of DSRs are periodically submitted to local heath bureaus and the central health information system maintained by NHFPC. For consistency, DSR has been standardized in accordance with the administrative requirement of the NHFPC (http://www.nhfpc.gov.cn/bgt/s6718/200905/40745.shtml). All DSR data, during the period from January 1, 2010 to December 31, 2012, were submitted to the monitoring group of NHFPC via a web portal (www.hqms.org.cn) in a standardized database format. In general, the DSR includes the following information on each hospitalization: basic demographics, admission and discharge dates, pre- and post-hospitalization diagnoses, treatments, outcome of hospitalizations, and financial costs. Electronic submission of DSR has been implemented since 2004. By 2010, all tertiary hospitals have routinely submitted their DSR to their regional health bureaus. While data contents in DSR are limited, their volume, timely submission, and complexity would qualify DSR database as a version of “Big Data” [12–16]. For international readers, all hospitals are classified into primary, secondary or tertiary levels, based on the healthcare delivery competence, with the tertiary level associated with the highest competence. Data made available for this research do not contain any personal identifier by National Institute of Hospital Administration, and hence personal consent is not required for this study with the exempt status. Given the primary emphasis on IM at discharge, the independent unit of the analysis is the hospitalization event from admission to discharge, rather than individual patient.

In this report, data from 43 tertiary general hospitals chosen to participate in a national pilot program that evaluates hospital performance under the direction of NHFPC are included. All DSRs without any personal identification information were provided to the authors for performing research. To protect privacy of individual hospitals, all hospitals were labeled as h###***, where the first three digits label region and the last three digits indicate hospital identification number within the region, without further disclosure. Inclusion of these hospitals is dictated by the availability and completeness of DSR data sets. The NHFPC has no influence on the design and conduct of this study.

### Hospitals under Study

Table 1 shows the key characteristics of all 43 hospitals under study as of year 2012, i.e., number of beds, number of doctors, number of nurses, and number of supporting staff. All numbers are either self-reported by hospital officials or extracted from associated health bureaus that oversee corresponding hospitals. There are three centrally controlled municipalities (Beijing, Shanghai and Chongqing; each with 13, 8 and 1 hospitals, respectively), where political and economic influence on China policies are equivalent or greater than, provincial governments. The remaining 21 hospitals distribute across 8 provinces: Gungdong, Hubei, Hunan, Jilin, Shangdon, Shannxi, Sichuan and Zhejiang. Note that these hospitals and their locations are not randomly chosen and should not be treated as representative populations in a strict scientific sense.

**Table 1:** Descriptive statistics of all 43 hospitals in this study (region, number of beds, number of doctors, number of nurses and number of staff) and numbers of hospitalizations across year 2010, 2011 and 2012.

Hospital	Provinces/	Numbers of				Hospitalizations in Year		
	Cities	Beds	Doctors	Nurses	Staff	2010	2011	2012
h011001	Beijing	1500	1015	1058	1567	37035	42121	49381
h011002	Beijing	1910	869	1342	784	55344	61139	70104
h011003	Beijing	1498	896	896	662	67343	65069	70400
h011004	Beijing	1500	822	1412	899	45638	52661	62515
h011005	Beijing	1448	842	1556	1147	47276	52190	58074
h011006	Beijing	1500	640	1038	984	29094	33012	38943
h011007	Beijing	950	684	689	681	26601	27605	35321
h011008	Beijing	860	896	1269	1265	44061	48245	56381
h011009	Beijing	2479	1112	1500	1388	59835	68135	63961
h011010	Beijing	981	699	1075	913	36604	39573	41664
h011011	Beijing	1100	634	1021	1227	21461	25921	29585
h011012	Beijing	1256	805	1258	891	31437	40002	47034
h011013	Beijing	1500	873	1402	1252	38429	40260	44825
h022001	Jilin	2303	940	1424	1695	62864	73602	84565
h022002	Jilin	3257	1287	662	578	94780	110132	131367
h022003	Jilin	3288	863	1305	1665	50887	82802	91603
h031001	Shanghai	1088	788	1147	884	50887	57610	62782
h031002	Shanghai	1700	909	1228	838	69020	75601	79531
h031003	Shanghai	2000	761	970	809	70940	73919	76720
h031004	Shanghai	1019	737	946	666	40894	45004	51653
h031005	Shanghai	1382	852	1437	903	65959	69008	79705
h031006	Shanghai	1950	734	1197	838	66134	75227	81517
h031007	Shanghai	2000	761	934	715	67225	70643	83226
h031008	Shanghai	1800	958	1537	1049	74793	77702	83506
h033001	Zhejiang	1900	814	1361	966	68143	76589	87886
h033002	Zhejiang	2500	1161	1884	1118	66149	74687	82951
h033003	Zhejiang	2400	656	1165	919	49461	55273	64296
h037001	Shandong	700	562	772	677	23056	23866	28967
h037002	Shandong	3000	922	2083	1474	59537	69919	99487
h042001	Hubei	500	171	314	233	5550	5715	6305
h042002	Hubei	4000	1133	2653	2156	87233	94479	122987
h042003	Hubei	4600	1270	2490	1430	84331	90559	105944
h043001	Hu’nan	3500	999	1811	722	76279	82948	92183
h043002	Hu’nan	1800	583	1074	903	56399	60872	67454
h043003	Hu’nan	3500	528	1957	1510	70879	86597	97756
h044001	Guangdong	2729	1155	2049	1830	78654	86598	93230
h044002	Guangdong	2200	888	1394	1237	37185	41512	52802
h044003	Guangdong	2548	1169	2021	1304	76529	80811	70462
h044004	Guangdong	2140	727	1090	1203	47757	53788	58568
h050001	Chongqing	3200	661	1952	1641	75444	84423	92802
h051001	Sichuan	4300	1046	2608	3027	141995	155609	160770
h061001	Shanxi	1700	577	1174	916	35100	44738	62371
h061002	Shanxi	2433	871	1671	1079	67885	76169	88982

### Inpatient Mortality

IM is computed as the total number of reported hospital deaths on DSR at discharge over the total number of discharges during the same period of time. Note that the total IM, for the entire hospital, is computed over all discharges, except for newborns. In this report, IM is used as a surrogate measure of the quality of healthcare delivery organization, per established standard by Agency for Healthcare Research and Quality (http://www.qualityindicators.ahrq.gov/Downloads/Modules/IQI/V31/iqi_guide_v31.pdf) and has been used in hospital evaluations[5, 6]. Admittedly, naïve use of IM as the sole indicator for healthcare quality is grossly inadequate[8–11]. In the current manuscript, we choose to use IM to measure healthcare quality, because of its accuracy, objectivity and comparability with reported IM from other countries. In fact, this indicator has been proposed as a measurement of healthcare quality, in evaluating hospital capability (2014 draft report on “standards for measuring healthcare delivery capabilities for tertiary general hospitals”) by NHFPC. The agency is currently placing the document in the government website (http://www.chinapop.gov.cn/zhuzhan/zqyj/201407/7a269c0f4ff34774b57505188edeb3b4.shtml), and is seeking public comment. As expected, blind use of IM for measuring healthcare capability in China is not without controversy (see debates by prominent healthcare professionals) http://www.sinohealth.com/2014/0728/6469.shtml) (also see Discussion below).

### Classification of Major Diseases by ICD-10

The DSR reported one primary diagnosis, along with seven secondary diagnoses, for each discharge, and all diagnoses are coded by ICD-10-CM. Under the leadership of NHFPC, all tertiary hospitals have adopted ICD-10-CM system to code diagnoses in their electronic medical record system and the DSR database. In a quality control study on DSR from all tertiary hospitals of Beijing, the accuracy in classifying major diseases (by first three digits) approached 100%, while the overall accuracy with all six digits was around 95% (personal communications). In China, the major factor affecting coding quality is the experiences of coders, rather than financial incentives that affect coding accuracy found in western countries. To focus the analysis presented in this report, five major diseases have been selected: myocardial infarction (ICD-10-CM codes are I21 and I22), pneumonia (J98.402, J10.0, J11.0, J12-16, J17 and J18), cerebral hemorrhage (I60, I61 and I62), cerebral infarction (I63) and traumatic craniocerebral injury (S06) (Table 2). These five diseases were chosen due to frequency, high clinical relevance and relatively higher reliable coding based on experiences.

**Table 2:** Distributions of patient's gender, age, major disease and major surgical treatments in this study population who receive hospitalizations in one of 43 hospitals during 2010 to 2012. Other total counts, remaining numbers are row percentages within each year.

Variable	Description	2010	2011	2012
**Total (x100,000)**		24.62	27.51	31.11
**Gender**	Female	49.57	49.65	50.06
	Male	50.43	50.35	49.94
**Age**	0–1	3.19	3.07	3.14
	2–6	3.11	3.13	3.05
	7–18	4.87	4.62	4.49
	19–29	12.04	11.67	11.29
	30–49	29.74	30.02	30.04
	50–64	28.80	29.50	30.01
	65–74	13.38	13.37	13.49
	≥75	4.87	4.62	4.49
**Major Disases**	Myocardial infarction	0.64	0.65	0.67
	Pneumonia	1.97	1.76	1.76
	Cerebral hemorrhage	0.85	0.80	0.76
	Cerebral infarction	1.80	1.73	1.73
	Traumatic craniocerebral injury	0.50	0.46	0.40
**Major Surgeries**	Coronary artery bypass graft	0.29	0.28	0.30
	Percutaneous coronary intervention	1.45	1.51	1.49
	Clearance of intracerebral hematoma	0.23	0.22	0.19
	Heart valve replacement	0.43	0.40	0.39
	Hip and knee joint substitution	0.53	0.57	0.62
	Malignant tumor surgery	1.78	1.84	1.83

### Classification of Major Surgeries by ICD-9.33

The DSR documented all surgical procedures via ICD-9-CM-3. In China, one version ICD-9-CM-3 is known as the national version, adopted for majority of hospitals, and another version modified by Beijing Health Bureau is used by others. Some cities/provinces are making minor local modifications for diagnoses, while corresponding diagnoses in Chinese are consistent across hospitals. Differences between these two ICD-9-CM versions are mostly in last three digits. For the purpose of this paper, we select six major surgeries, focusing on consistent diagnoses in Chinese and allowing some local variations to ICD-9-CM: coronary artery bypass graft (ICD9 code is 36.1), percutaneous coronary intervention (36.06, 36.07 or 00.66), clearance of intracerebral hematoma (01.24013, 01.31004, 01.39009, 01.39011, and several local modifications), heart valve replacement (35.2), hip and knee joint substitution (81.51–81.55 and several local modifications) and malignant tumor surgery (C16, C18, C20, C22, C34 and C64)(Table 2). All of these surgeries are routinely performed in most of tertiary general hospitals, and successes of performing these surgeries represent, to some degree, clinical competence. These surgeries are often cited by NHFPC to represent the overall level of clinical competence in a hospital.

### Statistical Analysis

Following the established data quality control process for the purpose of monitoring hospital performance, we compute frequencies of discharges by hospital, by year, by five major diseases, and by six major surgeries, using SAS 9.4 (www.sas.com). Also, we visualize IM in year 2010 and their trends to 2011 and to 2012 by using R3.02 (www.r-project.org), the plot of which is known as a point-direction figure. P-values for evaluating temporal trends of inpatient moralities for individual hospitals are computed by a single degree of freedom chi-square test as being implemented in prop.trend.test() function in R[17]. To assess any change of IM from year 2010 to 2011 and from year 2010 to year 2012, we use the binomial regression, embedded in the generalized linear model, to compute odds ratio (OR), Z-score and its p-value, after adjusting for hospital-specific heterogeneity[18]. Adjusted covariates in the analysis are 43 indicators for every hospitals under consideration, effectively adjusting for hospital-to-hospital heterogeneity. The statistical function, GLM, implemented in R3.02 is used to carry out all analyses. Finally, to effectively analyze this big data set, our analytic strategy has two stages: the first stage summarizes patient level data into hospital-specific summary frequencies, while the second stage analysis focuses temporal trends with appropriate adjustment to hospital-to-hospital heterogeneity.

## Results

### Infrastructures in Tertiary General Hospitals

Among these 43 tertiary general hospitals, numbers of hospital beds range from 500 beds to 4,600 beds, averaging size of 2,091 beds (Table 1). Likewise, the number of doctors, nurses and supporting staff varies widely. To account for variations in numbers of beds, we computed ratios of healthcare provider numbers over numbers of beds (see S1 Fig). The average ratio of doctors to beds is 0.47, ranging from 0.2 to 1.0 with a median of 0.35. The nurse to bed ratio averages 0.73, but varies widely from 0.2 to 1.4. The ratio of supporting staff to number of beds also varies widely, ranging from 0.5 to 3.5, with the average of 1.77.

### Increasing Hospitalization Capacity over Time

**Fig 1:**
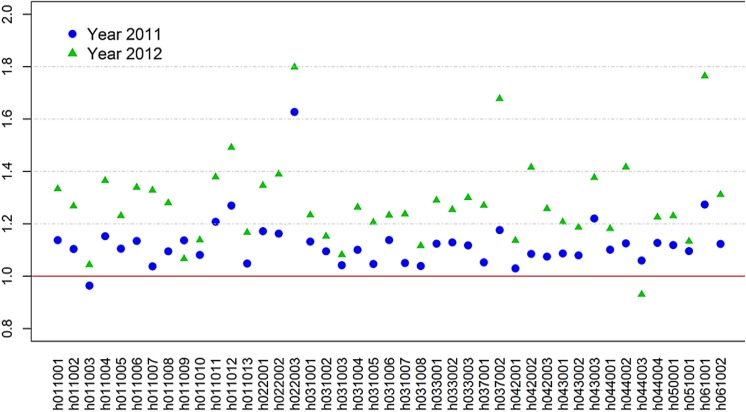
Computed ratios of discharge numbers in year 2011 (blue dots) and in year 2012 (green triangles) over discharge numbers in year 2010 (solid red line as the reference) across all 43 hospitals (organized by their geographic regions); nearly all ratios exceed one, corresponding to the increase of hospitalization capacity in these two years over the reference year.

Recent improvement in the Chinese economy and healthcare reform has gradually intensified the demand on healthcare system. In response to the increasing needs, all hospitals are steadily increasing their hospitalization capacity. Table 1 lists numbers of hospital discharges across all 43 hospitals, in year 2010, 2011 and 2012. Nearly all hospitals except for two (h011003 in year 2011 and h044003 in year 2012) experience substantial increases of discharges over these two years. To assist visual inspection, we compute ratios of total discharges in year 2011 over those in year 2010, and similar ratios for year 2012 (Fig 1). With the red line as the reference, nearly all hospitals increase their capacities from year 2010 to 2011 and to 2012, with average ratios 1.12 and 1.28, respectively.

### Basic Characteristics of Study Population

This study utilizes DSR databases from 43 hospitals, distributed across 3 major municipalities and 8 provinces in China, and captures a total of over 8 million discharges (Table 2). The numbers of discharges have been steadily increasing from 2.5 to 2.8 and 3.1 millions between 2010, 2011 and 2012, respectively. Female to male ratios, in receiving hospitalizations, are largely around 50%. Across all age groups, the proportions of discharges are stable, except for steady increase among subjects 50 to 64 year old. Among five major diseases, it is particularly noteworthy that discharges associated with cerebral hemorrhage and traumatic craniocerebral injury have steadily decreased each year, while discharges with myocardial infarction and cerebral infarction experienced a major reduction in year 2011. In contrast, surgery-related hospitalizations are largely stable, except hip and knee joint replacement which has been on the rise over these three years.

### Overall Inpatient Mortality

**Fig 2:**
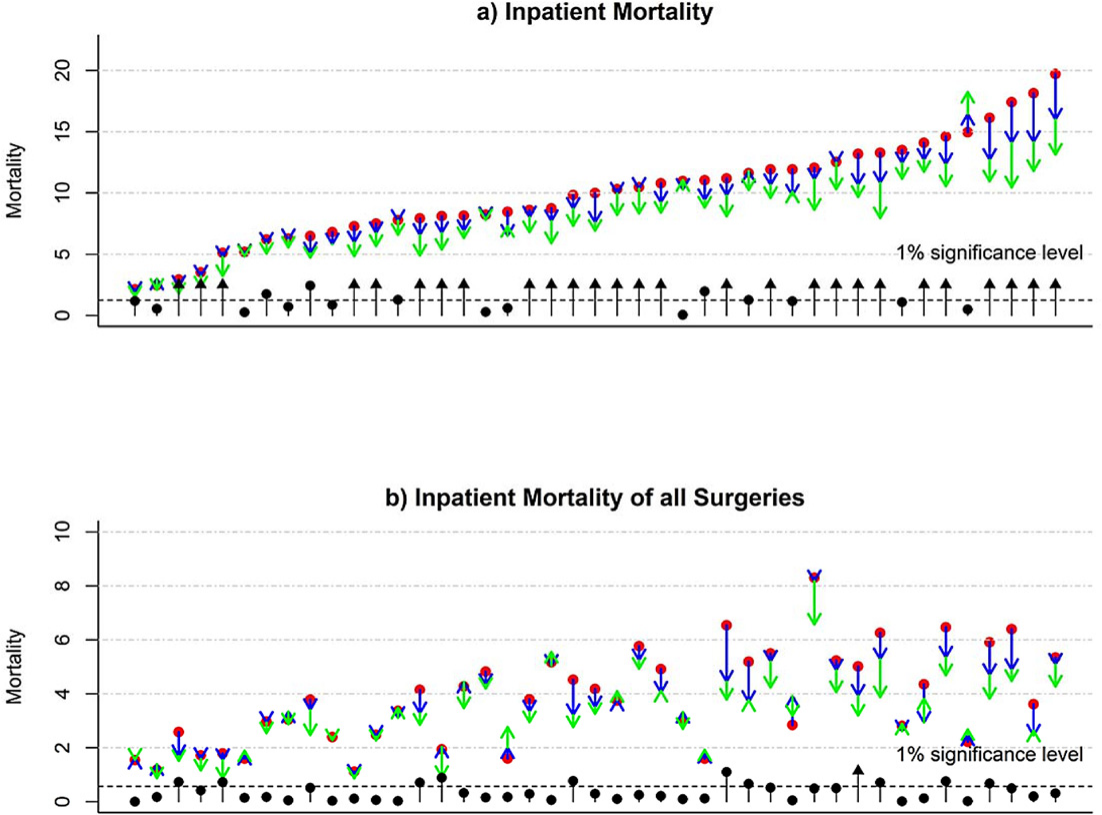
“Point-Direction” plots show inpatient mortality (multiplied by 1000, i.e., per thousand) in year 2010 (red dots) and their directional changes in year 2011 (blue arrow) and in year 2012 (green arrow) across all 43 hospitals: a) the overall inpatient mortality, and b) inpatient mortality of all surgeries. To represent statistical significances on temporal changes, computed p-values are transformed to logarithmic scale (-log_10_(p-value)) and are shown either as a black dot (actual p-value) or black arrow (actual p-value is less than 10^−4^).

Overall IM steadily declined by OR = 0.883 (p-value <0.001) in 2011 and further by OR = 0.766 (p-value < 0.001) in 2012, compared with IM in 2010 (the first row in Table 3). The variation in IM observed likely reflects the inclusion of 43 hospitals from diverse regions of China. Fig 2A shows hospital-specific IM sorted by IM for 2010. IM ranges from 2.10 ‰ (per thousand discharges) in hospital h043002 to 19.7‰ in hospital h011002. For individual hospitals, we use the “point-arrow” to represent the baseline IM in year 2010 (red dot) and its increments in year 2011 (green arrow) and 2012 (blue arrow). Clearly, the majority of hospitals demonstrate progressively declining IM. P-values (on a minus logarithmic scale, e.g., -log_10_(p-value) = 2 for p-value = 0.01) shown by black dots, with most exceeding the significance level of 5% (black dashed line). Despite the overall improvement in IM, one hospital (h042001) appears to show a non-significant increase in IM. Of note, hospitals with relatively high IM in 2010, tend to have greater improvement in reducing IM in 2011 and 2012, e.g., last four hospitals (h011010, h011012, h011011 and h011002). Interestingly, all four hospitals are located in Beijing.

**Table 3:** Comparison of inpatient mortality from year 2011 and 2012 with that in year 2010 (reference): Odds ratios (OR) quantify the change of associated mortality rates, Z-scores quantify signal to noise ratios, and p-values quantify statistical significance. All analyses are adjusted for hospital-specific heterogeneity and exclude those hospitals if associated discharges are less than 100.

	Year 2011			Year 2012		
	OR	Z-score	P-value	OR	Z-score	P-value
**Overall inpatient mortality**	0.883	-13.310	<0.001	0.766	-28.401	<0.001
**Inpatient mortality of all surgeries**	0.890	-5.213	<0.001	0.793	-10.380	<0.001
**Major diseases**						
Myocardial infarction	0.910	-2.004	0.045	0.793	-4.957	<0.001
Pneumonia	1.003	0.069	0.945	0.996	-0.075	0.940
Cerebral hemorrhage	0.832	-4.571	<0.001	0.764	-6.650	<0.001
Cerebral infarction	0.848	-3.431	0.001	0.755	-5.813	<0.001
Traumatic craniocerebral injury	0.953	-0.858	0.391	0.941	-1.063	0.288
**Major surgeries**						
Coronary artery bypass graft	0.950	-0.424	0.672	0.938	-0.551	0.581
Percutaneous coronary intervention	0.959	-0.486	0.627	0.800	-2.527	0.012
Clearance of cerebral hematoma	0.992	-0.118	0.906	0.861	-1.959	0.050
Heart valve replacement surgery	0.861	-1.456	0.145	0.889	-1.177	0.239
Hip and knee replacement surgery	0.904	-0.416	0.678	0.704	-1.428	0.153
Common malignancy elective surgery	0.795	-2.869	0.004	0.732	-3.916	0.000

### Inpatient Mortality of All Surgery-Related Hospitalizations

**Fig 3:**
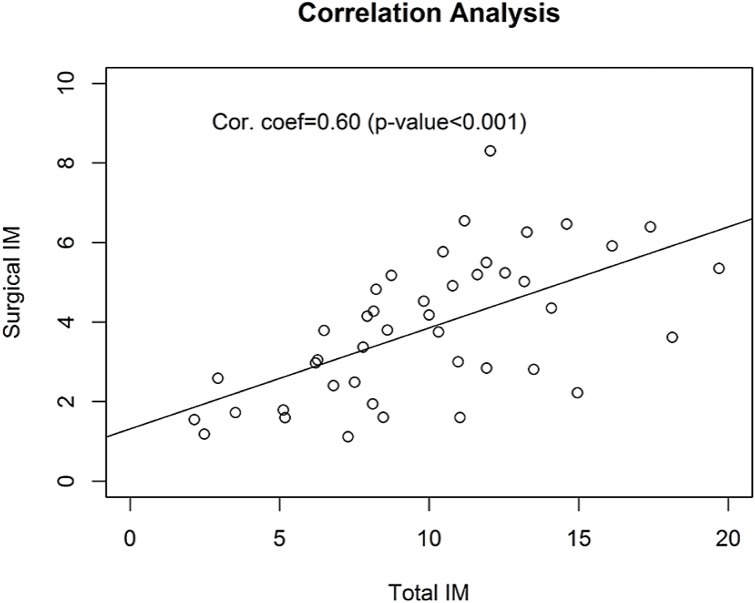
Correlation analysis of estimated inpatient mortalities of all surgeries (per thousand) with the overall inpatient mortalities (per thousand) over all 43 hospitals. While their correlation coefficient at 0.60 (p-value <0.001) is statistically significant, there are hospitals with modest overall inpatient mortality but very high surgery-related inpatient mortality.

Surgieries are probably the most important activities in demonstrating healthcare delivery capability, and IM associated with surgery is indicative of the overall healthcare quality of healthcare organizations. Compared with IM for all surgery in 2010, surgical IM in 2011 and 2012 are steadily improving with OR = 0.890 and 0.793 (p-values<0.001), after adjusting for hospital-related heterogeneity (Table 3). To examine hospital-specific and surgery-specific IM, we use a “point-arrow” plot to display IM following the same order of hospitals presented for overall IM (Fig 2B). Although surgical IM is strongly correlated with the total IM (correlation coefficient = 0.60, p-value < 0.001) (Fig 3), surgical IM across hospitals are not entirely synchronized with corresponding total IM. For example, hospital h051001, with relatively high overall IM, appears to have modest surgery-related IM, with reductions in surgical IM from 2010 to 2011 and further reductions in 2012 (p-value<0.001), bringing surgical IM down from approximately 5‰ in 2010 to 3 ‰ in 2012. On the other hand, there are also hospitals that show no improvement in surgical IM over these three years, for example, h009011 or h022001. To the contrary, one of hospitals (h061001) appears to have a non-significant increase in surgical IM in year 2012.

### Inpatient Mortalities of Five Major Diseases

As noted above, we chose IM associated with five major diseases to evaluate disease-specific healthcare quality, based on annual statistics reported by NHFPC (Table 2). Despite relatively high occurrences, some hospitals specialize in certain diseases, and may have relatively fewer patients in others. In this evaluation, entries associated with hospitals and years are excluded if the corresponding number of discharges were fewer than 100. Some hospitals are excluded entirely from the analysis, for this reason alone. After adjusting heterogeneity from hospital to hospital, Table 3 compares disease-specific IM in 2011 and 2012 with that in 2010. IM for myocardial infarction appears to be steadily declining from OR = 0.910 (p-value = 0.045) in 2011 to OR = 0.793 (p-value<0.001) in 2012. Similarly, IM of treating cerebral hemorrhage and cerebral infarction have also undergone significant improvement (p-values <0.001). Nevertheless, as shown in S1 Fig, changes in hospital-specific IM are not uniform. Some hospitals appear to have made major progress in reducing the associated IM while some other hospitals have made no progress.

In contrast with these three diseases, IM associated with pneumonia remains largely unchanged in 2011 and 2012 (OR = 1.003 and 0.996, p-values = 0.945 and 0.940, respectively) as does IM associated with traumatic craniocerebral injury (OR = 0.953 and 0.941, p-values = 0.391 and 0.288, respectively). Again, temporal trends in hospital-specific IM appear to vary considerably (S1 Fig). IM appears to be largely unchanged over these three years in some hospitals, while several hospitals have experienced increased IM.

### Inpatient Mortalities of Six Major Surgeries

As surgical capability is often a key criterion in measuring clinical competence of tertiary general hospitals, surgical IM are inevitably essential in measuring healthcare quality of hospitals. While overall surgical IM has been steadily declining, six common and clinically important surgeries were selected to examine surgery-specific IM. Among these six surgeries, a consistent and continuous decline of IM associated with elective surgery for malignancy has been observed (OR = 0.795 and 0.732 with p-values = 0.004 and <0.001, over year 2011 and 2012) (Table 3). It is important to note that several hospitals, with relatively high IM, have demonstrated major reductions in surgical IM in either 2011 or 2012, or both. Their continuing improvements contribute to the overall reduction of the IM (S2 Fig). Most other hospitals have had relatively stable IM, some of which are at relatively low levels.

Four major surgeries (percutaneous coronary intervention, clearance of cerebral hematoma, heart valve replacement surgery, and hip and knee replacement surgery) have experienced gradual reductions in IM in year 2012 alone or in both years. Although some have failed to reach statistical significance, the small numbers make interpretation difficult. As expected, IM and their temporal trends vary substantially across all hospitals. One notable hospital (h061002) has very high IM associated with hip and knee replacement surgery (~27 ‰) in year 2010, but then decreases to zero in 2011 and 2012. While perhaps encouraging, such a drastic change would also be a cause for further investigation.

Coronary artery bypass graft (CABG) surgery is among most challenging surgical procedures. Between 2010 and 2012, the associated CABG IM was largely unchanged (OR = 0.950 and 0.938 with p-values = 0.672 and 0.581 in year 2011 and 2012, respectively). Importantly, as shown in S2 Fig, only 15 hospitals performed more than 100 CABG surgeries in any given year. Among them, one hospital (h033002) operated on 93, 112 and 126 patients in year 2010, 2011 and 2012, respectively. Regardless of their IM in year 2010, most hospitals have not demonstrated much improvement with respect to IM.

## Discussion

Using IM as the primary endpoint, we use this “big data” set of over 8 million DSRs, to assess evolving healthcare quality in 43 hospitals in China from 3 major cities and 8 provinces, from year 2010 to year 2011 and to year 2012. In addition to assessing overall healthcare quality, we have investigated IM associated with five major diseases (myocardial infarction, pneumonia, cerebral hemorrhage, cerebral infarction and traumatic craniocerebral injury) as well as six major surgical procedures (coronary artery bypass graft, percutaneous coronary intervention, clearance of intracerebral hematoma, heart valve replacement, hip and knee joint replacement, and malignant tumor surgery). The analysis presented here suggests that while hospitalizing 12% and 14% more patients from year 2010 to 2011 and from year 2011 to 2012, respectively, the overall healthcare quality in tertiary general hospitals of China has made incremental progress. The increase in discharges is largely consistent with the increasing demand on healthcare systems due, in part, to better insurance coverage and improving personal financial status. Fortunately, this increasing demand appears to be matched by improving healthcare quality.

Examining hospital-specific IM over all hospitalizations as well as all surgeries, the results presented here also show substantial variation from hospital to hospital, which is often overlooked by simple summary statistics. While the presence of these heterogeneity calls in the question the validity of aggregate IM estimates, such variation has identified opportunities for many of these hospitals to improve healthcare quality. For example, the four hospitals with lowest overall IM (h043002, h033003, h04303 and h033001) have kept consistently high healthcare quality throughout all three years, with the IM of major diseases and major surgeries generally better than most other hospitals. Hence, these hospitals may have unique expertise in controlling IM, and their experiences may be valuable for other hospitals. In contrast, four hospitals with highest overall IM in year 2010 (h011010, h011012, h011011 and h011002) are among those that have greatest improvements in healthcare quality over these three year period. Again, their experience in reducing IM would also be valuable lessons for other hospitals. Indeed, our analysis has identified a few hospitals that have limited improvement in healthcare quality or even suffer from deteriorating IM. Such results could prompt physicians and hospital leaders to undertake their own investigation in order to find a root cause and attempt to improve healthcare quality.

One approach to identify a root cause is to study variations of hospital-specific IMs and their associations with hospital-specific characteristics, such as hospital infrastructures and patient compositions. For example, certain diseases or surgical treatments are associated with relatively high IM. Hence, compositions of patients, with respect to diseases and treatments, could alter IM. In many cases, high IM may well be justified, because of unique patient compositions. Meanwhile, we have presented summary statistics on personnel structures within hospitals (S1 Fig). Besides shedding light on the hospital structure of some best hospitals in China, these basic variables may also affect IM of hospitals. Identification of such factors could provide directions for hospital leaders to act upon, as an effort to reduce IM.

Another interesting dimension of observed IM across these hospitals is associated with the geography. Beijing, being the capital city, is known for the concentration of top tertiary hospitals in China, with support from prestigious universities, local government and various ministries of the central government, such as NHFPC, Ministry of Defense, Ministry of Transportation, etc. Likewise, Shanghai is known for hosting some of best physicians and hospitals in China. It is, therefore, rather surprising that the top four hospitals, with lowest IM, are not from Beijing or Shanghai. In fact, four hospitals from Beijing are among those with highest IM, even after their remarkable improvements in these three years. Meanwhile, those highly reputable hospitals in Shanghai appear to have IMs distributed around median IM. Clearly, referral patterns to hospitals across regions are quite different and it is likely that hospitals in Beijing and Shanghai admit patients with more complex diseases than hospitals in other cities/provinces. Hence profiles of co-morbidities in these Beijing and Shanghai hospitals may be very different from those elsewhere. Before reaching any definitive conclusion on ranking healthcare quality of these hospitals, therefore, more research must be undertaken with detailed information beyond the DSR.

Besides hospital-specific heterogeneity, our analysis has examined IM specific to five major diseases and to six major surgical procedures. While IM of treating three major diseases has substantially decreased in recent years, IM of treating pneumonia and traumatic craniocerebral injury appears to be essentially unchanged. It is recognized that relatively high IM for these two diseases may be associated with unique clinical characteristics that may complicate treatment. Nevertheless, the identification of these two diseases and potentially others may help clinical investigators to focus future interventions on these diseases. With regard to the six surgical procedures, all seem to have gradually improved healthcare quality. However, both coronary artery bypass graft and heart valve replacement surgery appear to associate with greater IM risk than other procedures, indicating the need for strategies that may reduce the associated IM.

To place above discussion of IM in an international context, we compare reported IM here with reported overall IM in US by Central Disease Control [19]. In US, overall IM is approximately 22.6‰ (= 715,000/35,100,000) in year 2010, which is slightly higher than the hospital h011002 with the highest IM among hospitals presented here. An erroneous interpretation of this comparison would be that all tertiary general hospitals considered had better healthcare quality than average US hospital. A major confounding factor is that reported IM in United States reflects the overall IM of all hospitals, as opposed to reported IM in top tertiary hospitals in China. Other confounding variables include differences in end-of-life arrangements, differences in financial incentives, and differences in hospice care. Nevertheless, the proximity of these estimated IMs also highlights similarity in healthcare outcome and quality between these drastically different healthcare systems. By more detailed comparisons and contrasts, we could identify their differences and similarities in these two healthcare systems, and could come up more innovative ways to improve patient-centered outcomes for both developing and developed countries.

It is also important to recognize intrinsic limitations of these data and assess their potential impact on the results and related conclusions. First, DSR is constructed for administrative purposes, i.e., facilitating hospital management by corresponding healthcare bureaus in local and central government and gathering information for evaluation purposes. While the DSR database provides adequate information on hospital structures (in term of hospitalization capacities, utilization of beds, types of diseases, numbers of surgeons, and types of surgeries, etc.) and also outcomes from hospitalizations (in term of admission and discharge status, costs, etc.), it includes limited clinical information or detailed information on healthcare delivery process. This limits our ability to identify specific causes that contribute to IM or many other aspects of healthcare quality. An ancillary limitation is that any adjusted analysis, such as computing expectations under certain confounders-adjusted regression, may yield less reliable summary results, such as risk-adjusted mortality rates (RAMRs). While this analysis includes 43 hospitals from 3 large cities and 8 provinces, the second limitation is that these hospitals cannot be considered as representative of all tertiary hospitals in China. Based on our familiarity with these 43 hospitals, it is reasonable to assert that these hospitals are among best tertiary general hospitals in China, with some over-representation of those from Beijing and Shanghai. Hence, it is reasonable to limit our statistical inference to hospitals located in these 11 regions and among top tertiary general hospitals, without generalizing our results and conclusions to all tertiary general hospitals in China. A third limitation is associated with use of IM as a measure for healthcare quality. As noted above, IM is certainly a useful index to measure healthcare quality, but is by no mean the only one[5]. In fact, IM may not capture accurately healthcare quality of a hospital when the case-mixture is not considered[8, 20]. It can also be confounded by length of stay and personal characteristics. Hence, when reaching our conclusion on healthcare quality, we need to be mindful of this limitation. In the future when electronic medical records are more readily available for big data analysis, we should seek additional indices to complement the IM in measuring healthcare quality. The fourth limitation associates with the fact that our analyses have not adjusted personal characteristics as possible confounding variables. The available DSR database is gathered for administrative purpose, and is certainly not so complete as typical research databases. Hence, the potential for detailed analyses, with adjusting for all possible confounders or building regression models for computing any expectations, is somewhat limited. Hence, we intentionally restrict our study largely as descriptive study. The primary comparison is on temporal trends, while some on spatial variations, results from which tend to be more robust. Ultimately addressing healthcare quality questions raised here, it will be important to design “analytic studies” focusing on certain disease treatment with more carefully curated variables from a well-defined study population. Nevertheless, the descriptive result, obtained here, provides some basic “ground truth” that may stimulates future analytic studies. The fifth limitation to this analysis is associated with ignoring multiple discharges of same patients. While it is a standard practice to focus on DSR per discharge as an unit of the analysis, this treatment has ignored the fact that some patients may be re-admitted into hospitals within days (or within weeks). Some re-admissions are pre-arranged, while others may be influenced by insurance coverages. Typically, such an ignorance may lead an under-estimation of variability, but should not create biases in estimations. Given the nature of this largely descriptive analysis, it is unlikely that the “over-dispersion” may mis-lead the observations made above. Nevertheless, when diving into specific diseases or specific surgeries, we should correct this potential over-dispersion, so that the statistical inferences by p-values are trustworthy. Lastly, but not least, DSR database includes only discharge reports, without providing any post-hospitalization follow-up information, prohibiting observation of vital status after discharge. Yet, an ultimate measurement of healthcare quality is the recovery rate from hospitalization and/or survival status post-treatment. To overcome this intrinsic limitation of studying healthcare delivery quality, it is important to collect follow-up information, to supplement DSR, in such a way that one can evaluate more meaningful index, such as mortality within 30 day post hospitalization, than IM. In light of shifting emphasis on patient-centered outcome by China Healthcare Reform, promoting routine collection of follow-up information, at least for some major diseases or treatments, would be important and implementable policy.

The recent report on measuring healthcare capacity by NHFPC (noted in Method section) has promoted use of IM as a metric for healthcare quality. Nevertheless, it is important to be cautious about over-interpreting IM as a quality measure. First of all, it is essential that reported rates of IM are accurate and objective in order to minimize bias in reporting. With the modernization of electronic medical record systems and systems for generating and managing DSRs, all tertiary hospitals should automate the data capture and submission. Secondly, under the leadership of NHFPC, it may be necessary to initiate a national pilot project, assessing distributions of key indicators such as IM. For instance, 20 out of 43 hospitals have not met the proposed standard for IM of 0.8% in 2012. Likewise, none of 43 hospitals would meet the surgery-related IM standard of 0.14%. Thirdly, when the IM is considered a performance measurement, it is not ideal to set an arbitrary and absolute standard, such as all hospitals could exceed or no hospitals can meet. Instead, it is essential for the medical community to come up a consensus on a range of index values, which could be adjusted by multiple factors, such as referral patterns, severity of diseases, case-mixture, etc.

Current healthcare reform efforts in China are now in their sixth year. Most reform activities center on hospital organizations (i.e., structures) and fees and incentives (i.e., financial incentives). Increasingly, the reform emphasis is shifting toward healthcare organizations, fundamentally improving healthcare quality. As healthcare reform shifts its emphasis, criteria of evaluating the success of healthcare reform should be modified from “financially covering all patients to “patient-centered outcomes”, including patient satisfaction and quality of care. Ultimately, healthcare is intended to control patients’ illness and to return patients back to healthy and happy status physically and emotionally. To measure the success of the healthcare reform, big data, such as DSR database, can be extremely useful and can complement other indicators that measure patient satisfaction.

Before ending the discussion and reaching an overall conclusion, we would like to bring up a potentially controversial result relating to IM associated with acute myocardial infarction (AMI), which seems to conflict with conclusion of a recent report by Li et al [21]. While fully exploring this conflicting observation requires much more careful scrutiny and is beyond the aim of this paper, here we just highlight several key factors that may contribute to their differences. First of all, the sampling populations are rather different. Here we have chosen 43 tertiary general hospitals, representing some of best hospitals in China, and have used all of hospitalizations treating AMI. In contrast, Li et al. used a multi-staged sampling strategy, covering many hospitals and sampling only few patients from each hospital. Their result may be more representative of all hospitals at all levels. Secondly, our study focuses on the period of year 2010–2012, while Li et al. study collects data from year 2001, 2006 and 2011. Because of dramatic recent changes in China, this difference in study time frames could contribute to this conflicting result. Last but certainly not least, patient mixtures between these two studies are rather different; Li et al study uses exclusively ST-segment elevation myocardial infarction patients, versus all AMI patients in our study. Netherless, resolving the conflicting observations is important and requires further study.

In conclusion, using IM as a proxy, we have observed that healthcare qualities are highly variable across these best tertiary general hospitals in China, and that nearly all hospitals have continuously reducing IM over year 2010, 2011 and 2012. Improvements have been observed for several major diseases or surgeries. Collectively, these results support that the recent China healthcare reform has positive impact on healthcare quality in Chinese healthcare delivery system. As the healthcare reform continues, the emphasis may be shifted towards bridging gaps of healthcare qualities between hospitals, and towards sub-specialties in medicines. More broadly, strategies adopted by the China healthcare reform may be worthy careful studies and, with appropriate modifications, may guide other nations in designing their own healthcare reform strategies.

## Supporting Information

S1 Fig Distributions of four key hospital characteristics (number of beds, number of doctors per bed, number of nurses per bed, and number of supporting staff per bed) among selected 43 hospitals. (PNG)

S2 Fig “Point-Direction” plots show disease-specific inpatient mortality (per thousand) in year 2010 (red dots) and their directional changes in year 2011 (blue arrow) and in year 2012 (green arrow) across all 43 hospitals: a) myocardial infarction, b) pneumonia, c) cerebral hemorrhage, d) cerebral infarction, and e) traumatic craniocerebral injury. Points/arrows are not shown if underlying numbers of discharges are fewer than 100.
(BMP)

S3 Fig “Point-Direction” plots show surgery-specific inpatient mortality (per thousand) in year 2010 (red dots) and their directional changes in year 2011 (blue arrow) and in year 2012 (green arrow) across all 43 hospitals: a) coronary artery bypass graft, b) percutaneous coronary intervention, c) clearance of cerebral hematoma, d) heart value replacement surgery, e) hip and knee replacement surgery, and f) common malignancy elective surgery. Points/arrows are not shown if underlying numbers of discharges are fewer than 100.
(BMP)

## Abbreviations

DSR: Discharge Summary Report
HR: Healthcare reform
IM: Inpatient mortality
NHFPC: National Health and Family Planning Commission
OR: Odds ratio
95% CI: 95% Confidence interval
