# Supplementary figures and images for: Evolving Healthcare Quality in Top Tertiary General Hospitals in China during the China Healthcare Reform (2010–2012) from the Perspective of Inpatient Mortality
Source: PLoS One. 2015 Dec 1;10(12):e0140568. doi: 10.1371/journal.pone.0140568 (PMC4666409; doi:10.1371/journal.pone.0140568)

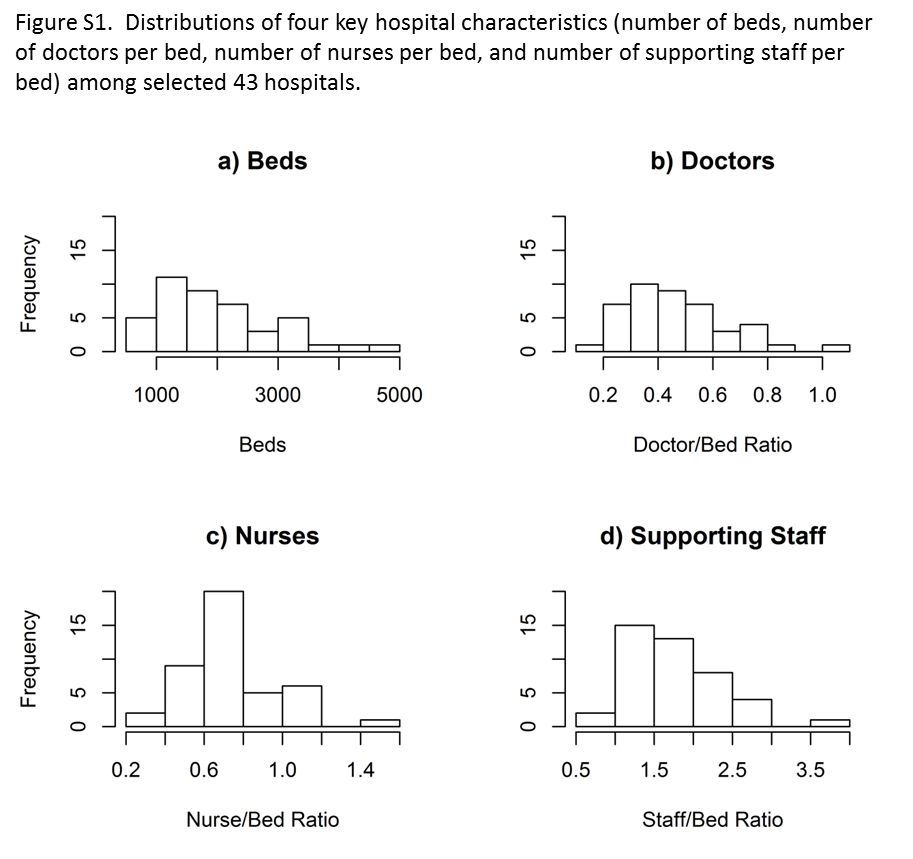

Supplement: S1 Fig — (PNG) [file pone.0140568.s001.png]

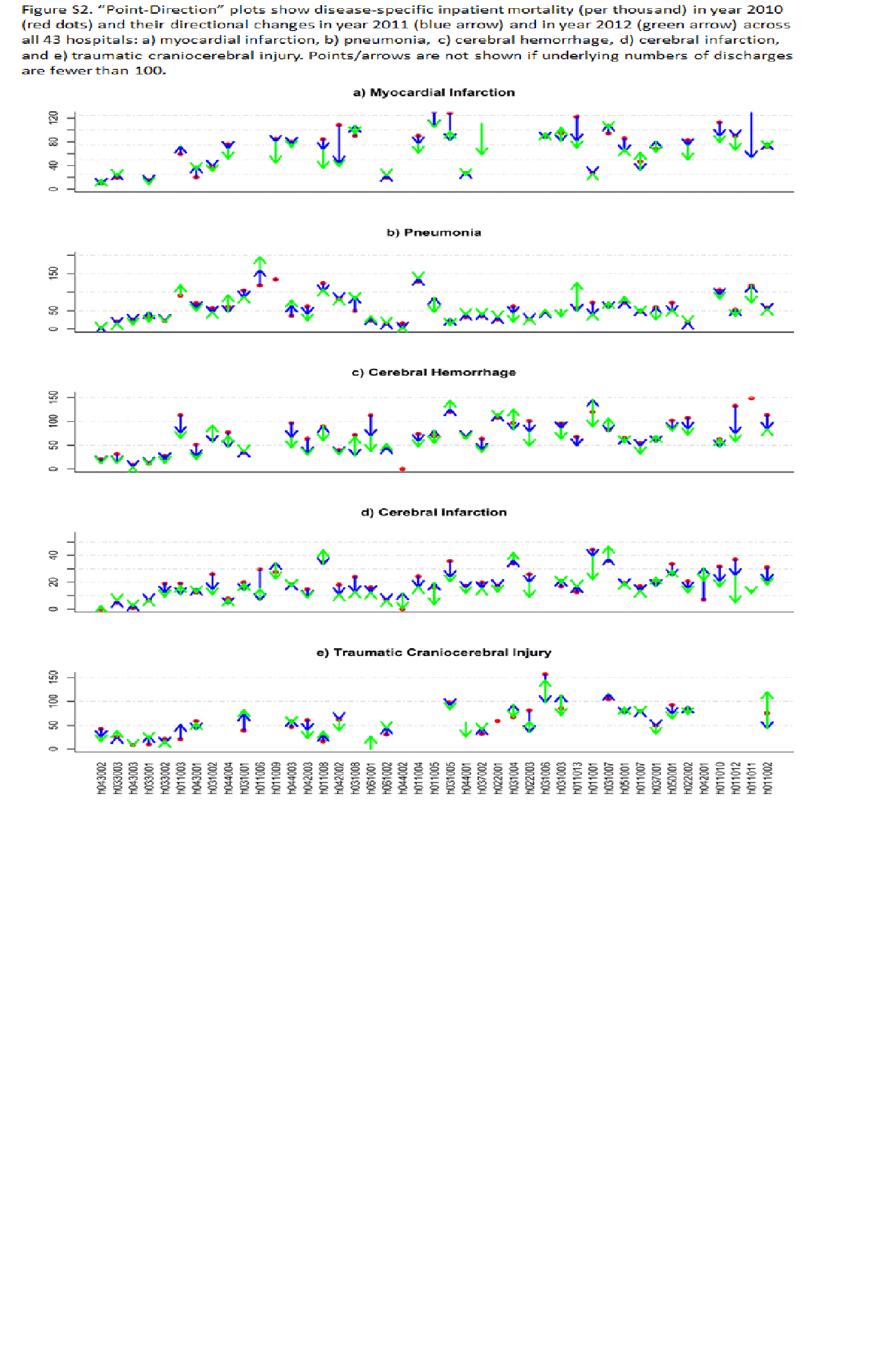

Supplement: S2 Fig — Points/arrows are not shown if underlying numbers of discharges are fewer than 100. (BMP) [file pone.0140568.s002.bmp]

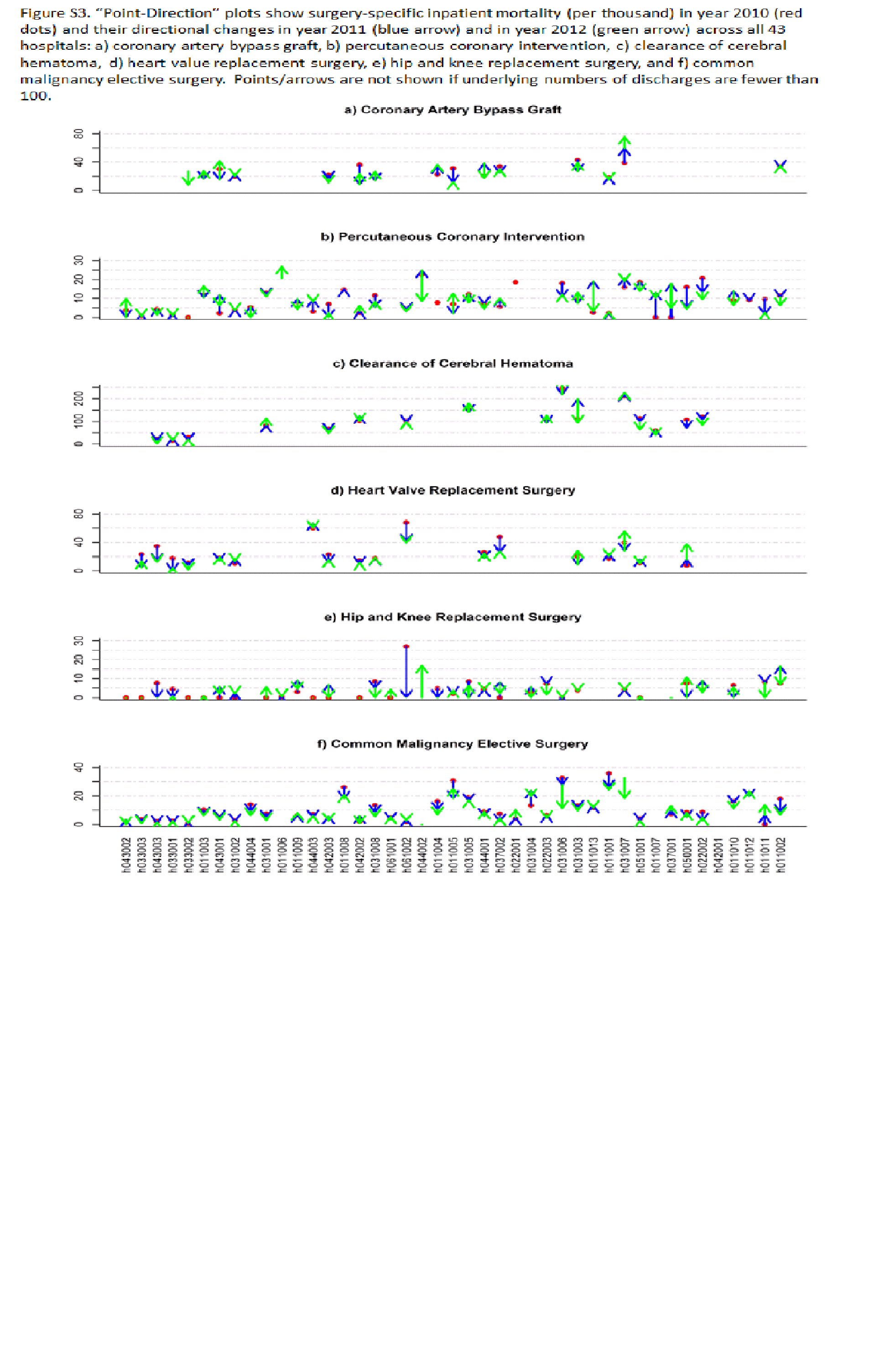

Supplement: S3 Fig — Points/arrows are not shown if underlying numbers of discharges are fewer than 100. (BMP) [file pone.0140568.s003.bmp]
